# Attitudes towards mental health, mental health research and digital interventions by young adults with type 1 diabetes: A qualitative analysis

**DOI:** 10.1111/hex.12662

**Published:** 2018-01-10

**Authors:** Janine Clarke, Judy Proudfoot, Veronica Vatiliotis, Charles Verge, Deborah J. Holmes‐Walker, Lesley Campbell, Kay Wilhelm, Catherine Moravac, Pillaveetil S. Indu, Madeleine Bridgett

**Affiliations:** ^1^ Black Dog Institute Randwick, Sydney NSW Australia; ^2^ UNSW Australia Sydney NSW Australia; ^3^ Sydney Children's Hospital Sydney NSW Australia; ^4^ School of Women's and Children's Health UNSW Australia Sydney NSW Australia; ^5^ Department of Diabetes & Endocrinology Westmead Hospital Westmead NSW Australia; ^6^ Department of Endocrinology St Vincent's Hospital Sydney NSW Australia; ^7^ Urban Mental Research Institute St Vincent's Hospital Sydney NSW Australia; ^8^ University of Toronto Toronto ON Canada; ^9^ Government Medical College Trivandrum Kerala India; ^10^ St James Hall Syndey Australia

**Keywords:** digital interventions, psychosocial support, type 1 diabetes

## Abstract

**Background:**

Young people with type 1 diabetes are at increased risk of mental disorders. Whereas treatment need is high, difficulty recruiting young people with type 1 diabetes into psychosocial studies complicates development, testing and dissemination of these interventions.

**Objective:**

Interviews with young adults with type 1 diabetes were conducted to examine attitudes towards mental health and mental health research, including barriers and motivators to participation in mental health studies and preferred sources of mental health support. The interviews were audio‐taped, transcribed and evaluated via thematic analysis.

**Setting and participants:**

Young adults with type 1 diabetes were recruited via social media channels of 3 advocacy organizations. A total of 31 young adults (26 females and 5 males) with an average age of 22 years were interviewed between October 2015 and January 2016.

**Results:**

Participants were largely unaware of their increased vulnerability to common mental health problems and knew little about mental health research. Major barriers to participation included perceived stigma and lifestyle issues and low levels of trust in researchers. Opportunities to connect with peers and help others were described as key motivators. Psychological distress was considered normal within the context of diabetes. A need for some level of human contact in receiving psychosocial support was expressed.

**Discussion and conclusion:**

Findings provide valuable insights into the complex dynamics of engaging young adults with type 1 diabetes in mental health studies. Interviewees provided practical suggestions to assist investigation and delivery of psychosocial interventions for this vulnerable group.

## 1 INTRODUCTION

Prevalence of mental disorders is greatest in adolescence and early adulthood,^1^ and adolescents and young adults with type 1 diabetes are an especially vulnerable group.^2^, ^3^ Daily self‐care of type 1 diabetes involves complex activities that are often highly intrusive into daily life including monitoring of blood glucose levels and hyperglycaemic and hypoglycaemic symptoms, assessing carbohydrate intake with insulin treatment (multiple injections or infusion from a pump); and meal‐to‐meal adjustment of insulin dose depending on diet and physical activity. How well young people cope with the emotional and behavioural challenges of type 1 diabetes, while at the same time negotiating the normative developmental transition to adulthood, has important implications for long‐term physical and mental health, quality of life and life expectancy.^4^, ^5^ Unfortunately, data suggest that many young adults with type 1 diabetes do not seek face‐to‐face support for their emotional health needs.^2^, ^6^

Despite a clear need, research into development, evaluation and dissemination of novel mental health interventions for young adults with type 1 diabetes is limited, possibly because of difficulties recruiting this group for psychosocial studies.^7^ Young adults, generally, are less likely to volunteer for psychosocial research for various pragmatic and lifestyle reasons including scheduling conflicts (due to work, education and other responsibilities), lack of interest and perceived personal irrelevance of the research focus and treatments.^7^, ^8^ Recruitment of young adult men is complicated further by high mental health stigma, inadequate knowledge of mental health signs and symptoms, and privacy and confidentiality concerns.^9^, ^10^, ^11^, ^12^ For researchers targeting young people with type 1 diabetes, low disease prevalence in the 15‐ to 24‐year‐age group (approximately 0.2%) is an additional recruitment barrier.^6^ Direct referral to psychosocial studies by health practitioners can be a valuable and cost‐effective method for recruiting people with medical conditions.^13^ However, low rates of attendance at diabetes clinics and medical appointments by young people with type 1 diabetes^14^ mean that even this targeted recruitment approach is likely to yield only a small group of eligible candidates.

Comfort, confidence, proficiency and engagement with digital technologies are frequently offered as reasons for using social media channels and Internet outreach to access young adult candidates for research studies.^15^, ^16^, ^17^, ^18^, ^19^ At the same time, evidence suggests that recruitment of young adults with medical conditions may still benefit from adjunctive local strategies encouraging direct referral via health‐care providers in specialist programs, clinics and services targeting young people.^15^ We adopted both recruitment methods in a recent controlled study of a broadly available, mobile phone and web‐based self‐help program for reducing mental health symptoms and improving positive well‐being in young people (aged 16‐25 years) with type 1 diabetes. Specifically, we liaised with stakeholder and advocacy groups to develop trial messaging for dissemination via a range of social media outlets, including Facebook, Twitter and appropriate websites. At the same time, young people attending type 1 diabetes “transition” services (ie, supporting transition from paediatric to adult health services) were approached directly by clinic staff during routine follow‐up appointments. Despite an exhaustive and laborious 12‐month recruitment campaign involving more than 40 different social media and Internet options and ongoing direct referral, we were unable to enrol sufficient young people into our study.

Understanding how best to increase recruitment into clinical research trials is gaining increased scientific interest,^20^, ^21^, ^22^ with research suggesting that effective strategies are unlikely to be universal, but to vary as a function of the target group and study methodology.^23^ At the same time, identifying the factors impacting research participation decision making seems critical for designing interventions aimed at optimizing trial recruitment.^20^ For this reason, and in the light of our recruitment experience, we decided to examine the factors influencing decisions made by young adults with type 1 diabetes in regard to their mental health and participation in mental health research.

Specifically, the study used qualitative methods to obtain an in‐depth understanding of:

- the attitudes and feelings of young adults with type 1 diabetes towards mental health and mental health research and
- the enthusiasm of this cohort for digital mental health interventions.

Our aim was to gain vital information to inform the design, promotion and recruitment methods of future studies of psychosocial interventions for young adults with type 1 diabetes.

## 2 METHODS

### 2.1 Participants

In‐depth interviews and focus group discussions were conducted with young Australian adults (18‐30 years) with type 1 diabetes between October 2015 and January 2016. Participants responded by email to advertisements placed on Facebook and Twitter, announcements on websites of local diabetes advocacy agencies and/or were referred by staff of transition services in NSW.

Potential candidates were contacted by phone for screening by one of 2 researchers who are clinically trained (VV, JC). Candidates were eligible to take part in the study if they obtained depressive symptoms scores in the mild‐to‐moderately severe range (ie scores 5‐19 inclusive) on the Patient Health Questionnaire—9 (PHQ‐9;^24^), and/or reported diabetes distress in the moderate range (ie scores greater than or equal to 3) on the Diabetes Distress Scale—2 (DDS‐2;^25^). Return of a signed consent form was also a criterion for eligibility. Potential candidates who scored in the severe range on the PHQ‐9 were recommended to seek face‐to‐face support and provided advice and information about self‐help resources and crisis mental health services.

A “sampling to saturation” recruitment method was used in which data collection continued until no new themes emerged from the interviews. Participants received a $40.00 gift voucher as appreciation for their time and contribution. The study was approved by the UNSW Sydney's (The University of New South Wales) Human Research Ethics Committee (HC15519).

### 2.2 Interviews

Interviews and focus groups were facilitated by 2 of the authors (JC, VV). They lasted between 30 and 70 minutes, were digitally recorded and transcribed verbatim. Interviews were semistructured and asked a range of open‐ended questions about the following:

- Attitudes of young adults with type 1 diabetes towards mental health and mental health research,
- Potential barriers to and motivators for participating in mental health research,
- Strategies for engaging young adults with type 1 diabetes in mental health research and
- Attractive sources of mental health support for young adults with type 1 diabetes.

### 2.3 Analysis

Data analysis was conducted primarily by one of the authors (CM) and was guided by Braun and Clarke's^26^ thematic analysis framework. This involved working sequentially through each of the transcripts, making notes about potential themes, key observations and highlighting each participant's key attitude, preference concerns and experiences. The transcripts were then reviewed a second time to ensure that nothing had been missed, and initial codes were discussed with authors JP and JC. NVivo 11 qualitative research software (QSR International Pty. Ltd., Victoria, Australia) was then used to facilitate analysis, and codes were then further grouped and organized. The organized themes and subthemes were then reviewed and agreed by CM, JP and JC.

## 3 RESULTS

### 3.1 Participants

As shown in Table 1, thirty‐one (31) young adults with type 1 diabetes were recruited for this study. The sample was predominantly female (n = 26) with an average age of 22 years (SD = 3 years) and an average age of diabetes onset of 13 years (SD = 6.5 years). Sixty‐five per cent (n = 20) of participants screened positive for diabetes distress, and the average score on the PHQ‐9 was in the “mild” range ($\overset{¯}{x}$ = 7.9; SD = 4.1). Only 5 participants had previously taken part in mental health research.

**Table 1:** Sample characteristics

Characteristic	
Mean age (SD)	22 (3)
Mean age of type 1 diabetes onset (SD)	13 (6.5)
Mean PHQ‐9 score (SD)	7.9 (4.1)
Female	26 (84%)
Previous participation in mental health research	5 (16%)
Positive screen for diabetes distress (>=3)	20 (65%)
PHQ‐9 classifications	
Normal	7 (23%)
Mild	17 (46%)
Moderate	8 (26%)
Moderately severe	2 (6%)

Two focus groups were conducted, each comprising 4 participants. The authors (JC and VV) each lead one focus group, with the other author observing to ensure consistency of approach. The remaining 23 participants were interviewed by one of the authors (either JC or VV).

### 3.2 Emergent themes and subthemes

#### 3.2.1 Attitudes towards mental health and mental health research

Table 2 summarizes the major themes and subthemes that were derived from participant responses regarding attitudes towards mental health. Almost all participants were unaware of the relevance of emotional well‐being for health outcomes and longevity in people with type 1 diabetes. Many described diabetes management as overly preoccupied with “numbers” (ie controlling blood glucose levels), with little attention to helping young adults “live” well with the disease.

**Table 2:** Emergent themes and subthemes: Part 1

Major themes	Common subthemes
Attitudes towards mental health	
Awareness of link between diabetes and emotional well‐being	Unaware Aware
Personal salience	Emotional well‐being not impacted
Help seeking	Already accessing emotional supports
Health‐care provider‐patient interactions	Little to no discussion about psychosocial issues
Mental health stigma	Fear of being “judged” Pressure to be “normal”
Potential barriers to participating in mental health research	
Participant lack of awareness	Of mental health research Of research opportunities
Distrust of research process	False claims Privacy and confidentiality concerns
Attitudes of health professionals	Emphasis on “numbers”
Practical and logistical barriers	Time Location
Emotional issues	Fear of getting labelled Denial Judgement Guilt
Potential motivators to participating in mental health research	
Information, information, information	Provide detailed information about the research, including research protocol and how findings will be used Raise awareness of health professionals
Altruism	Desire to help others
Meet our personal needs	Self‐knowledge Connection
Tangible incentives	Distinction between “self” and “others”

Some participants described raising emotional issues in the context of routine diabetes care, however, felt that their concerns had been either minimized or dismissed by their treating health professionals. As a consequence, participants were largely unaware of the increased vulnerability of young adults with type 1 diabetes to common mental health problems. Moreover, many participants considered mental health research as mostly irrelevant to their overall physical health.

And they pretty much told me over the course of a couple of days how to inject, how to read labels, how to manage the disease but not how to manage everything else. (Female, age 30, diagnosed at 29 years)

I was just told that it was all in your head. [ ] no one could recognise that diabetes affects mental health and mental health affects diabetes – it's like a dog chasing its tail. (Female, age 24, diagnosed 23 years)

Well specifically regarding mental health, this is the first I've heard of research into mental health conditions within type 1 diabetes. I guess it's always the medical aspects that I've seen. [] But I always feel like [ ] they're looking for other people to get the information from, not me personally. (Female, age 20, diagnosed at 12 months)

A small number of participants reported a personal history of depression and suggested that this increased the personal relevance and “appeal” of mental health studies.

I'd reach out immediately. I've had depression in the past [ ] I'd do anything to help lessen the blow. (Female, age 22, diagnosed at 20 years)

These young adults nevertheless expressed appreciation for the majority view that mental health difficulties are potentially isolating, stigmatizing and embarrassing, with involvement in mental health research having the potential to distinguish them further from their physically and psychologically “healthy” peers.

And it's just I kind of freak out about that, so I don't think about it very much because it scares me. (Female, age 22, diagnosed at 10 years)

Sometimes talking about mental health can be quite confronting and quite scary. (Female, age 20, diagnosed at 9 years)

[ ] when you've already got a chronic illness you're probably going ‘do I really want to be associating this with me as well and then I'll stand out more’, and that's not good when you're a young teenager. (Female, age 19, diagnosed at 14 years)

Even participants with no previous mental health diagnosis reported struggling from time to time with a broad range of emotional issues with varying levels of severity, including shame, guilt, fear, low motivation, loneliness, helplessness and hopelessness. Overwhelmingly, these difficulties were considered a normal response or “side‐effect” of living with a complex and demanding illness. Participants felt that it was inappropriate to “overpathologize” these experiences by applying diagnostic labels to them.

#### 3.2.2 Potential barriers to and motivators for participating in mental health research

Tables 2 also summarizes the major themes and subthemes that emerged from discussion of the potential barriers and facilitators to participation in mental health research.

By far, the greatest perceived barrier for participants was poor understanding of the research process generally. Specifically, most interviewees admitted to knowing little about the purpose of mental health research, the different types of research studies (eg single point vs longitudinal studies), who (organizations and individuals) conducts mental health research, for whom such research is relevant, and how research data are stored and used. Furthermore, many young adults claimed never to have heard about any opportunities to take part in mental health research.

We probably don't think about it that much [ ] I know I haven't really thought about being a part of it because the opportunity has never really come up for me. (Female, age 20, diagnosed at 3 years)

Lifestyle factors were also offered by many participants as potential barriers to research participation, especially time constraints. One participant had this to say:

Let us check in when we've got time. We all lead very busy lives and we may not be available at the same time every week, but even if it's in an online capacity – if you wanted to follow up with us, sometimes even just leaving us a message and letting us give you a ring back when we have a moment, is an easy way. (Female, age 24, diagnosed at 23 years)

[ ] so if there were giant bean bags and – for our age group I feel like you would think, oh, yeah, bean bag, go and sit on it and then everything just comes naturally. (Female, age 20, diagnosed at 11 years)

Ambivalence, confusion and scepticism about the intent and motivation of scientific research were identified as further barriers to participation. Participants explained that the perception of scientific research had been tarnished by what they considered false claims and unsubstantiated statements about potential new treatments and promises of a “cure” for type 1 diabetes, and the benefits of which they were unlikely to experience in their lifetime. As a consequence, there was reduced confidence in research and research outcomes, research was considered irrelevant, with some participants concerned about the potential for misuse of research data, for example provision of personal information to insurance companies.

I think a lot of young people and a lot of older people get so overwhelmed by all the research that's done. They don't really know what to focus on or what the relevant things are for them. Constantly having family or friends linked with research pages on Facebook, pages saying that they've made new discoveries into fake pancreases and all this, research that is not relevant. They think it's going to cure me – they mean well, and they want to help but there's just too much research out there. (Female, age 18, diagnosed at 15 years)

Yeah, I guess they don't really see any positive outcome to it. [] whenever I mention that I'm type 1 a lot of people say, “There's so many medical breakthroughs these days and research and there will be a cure before you know it. Don't worry about it,” sort of thing. Whereas it's easy to say that, in reality it's not that easy. So I think a lot of people wouldn't support it because they don't see a goal at the end of it. (Female, age 20, diagnosed at 19 years)

[ ] ran a story a couple of weeks back on “Drink this, it will cure your diabetes” I damn wish – but it's not the case. (Female, age 18, diagnosed at 15 years)

Participants also described a range of emotional issues as impediments to participation in mental health studies. For example, some expressed a reluctance to identify oneself as having diabetes or “denial” as important obstacles.

I think people just want to avoid it [ ] sometimes I just feel like I can live without paying attention to my diabetes, or just pretend it's not there. [ ] I think that's quite a common way of coping with it all when it becomes too much. I think you just want to [ ] live like everyone else, and doing the research forces you to think about it and [ ] people just don't want to do that. (Female, age 18, diagnosed at 7 years)

Participants spoke about the stigma of mental illness, concerns about stereotypical judgements and unpleasant labels as negatively impacting their willingness to engage in mental health research. Many experienced stigma as externally driven by society, while for some it was also perceived as self‐imposed. Many participants also reported feeling negatively judged by virtue of having a chronic illness. For these people, unwillingness to participate in mental health research was understood as a way to avoid the “double stigma” associated with having comorbid physical and mental health issues.

Not only is it the stigma from other people and from society about mental illness that really prevents young people talking about it – it's also the stigma they put on themselves. [ ] they can be really harsh on themselves, thinking ‘I'm failing and not doing what I'm supposed to be doing – it's all my fault’ [ ]. I think that would really stop them from thinking about it. (Female, age 18, diagnosed at 15 years)

Even just admitting that [ ] someone has anxiety or you need help, can be seen almost as a weakness. And if you already have a chronic illness [ ] you don't want to admit that to yourself. (Female, aged 18, diagnosed at 12 years)

When asked about the motivators for research participation, many participants emphasized that young adults with type 1 diabetes would be more likely volunteer if they were better informed about the research process, and more appreciative of the link between diabetes and mental health. Responses indicated young adults desire to fully appreciate the personal impact of the research (including time commitment, short‐ and long‐term benefits of participation, treatment and storage of their personal information) prior to volunteering. Another major theme reflected altruistic intentions to “make a contribution,” with several participants describing the opportunity to make a difference to the lives of other young adults as potentially a key motivator of research participation.

[ ] dealing with what I've gone through [ ] would make me want to help if I can. [ ] it wouldn't benefit me too much, but it would benefit people in the future. So, yeah, that would be my main incentive. (Female, age 25, diagnosed at 19 years)

[ ] having a sense for what we say and turning up will have an effect [ ] It's knowing what I had to say may have helped someone else. (Female, age 24, diagnosed at 23 years)

During the interviews, some participants reported that our study was their first opportunity to interact with similarly diagnosed peers. Thoughts were expressed about the potential for mental health research studies to provide young adults with type 1 diabetes opportunities to connect in ways that ensured their privacy, and the potential for this to motivate research involvement.

I just like talking about diabetes [ ] especially with other young people, because it makes me feel not as alone. (Female, age 20, diagnosed at 9 years)

Just mainly that it would be nice to see research lead to a social connection [ ] everything comes together. (Female, age 18, diagnosed at 12 years)

Participants generally disagreed that tangible rewards would encourage their participation in mental health research. Nevertheless, they felt that other young adults might be “incentivized” if the recompense offered satisfied either the basic (eg food and clothing) or recreational (eg music and movies) needs of young adults.

#### 3.2.3 Strategies for engaging young adults in mental health research

Common major themes and subthemes that emerged in participant responses to questions about how best to engage with young adults are shown in Table 3.

**Table 3:** Emergent themes and subthemes: Part 2

Major themes	Common subthemes
Engaging young adults with type 1 diabetes in mental health research	
Raise awareness	Raise awareness of the link between mental health and diabetes health in health professionals and young people Raise awareness in young people Explain the research process
Young adults’ needs are central in all aspects of the research process	Lifestyle Peer support Youth “culture” Involve young adults in protocol development
Involve research ambassadors	Use public figures to promote research
Targeted promotional campaigns are more effective	Engage with social media Partner with organizations/people that young people trust
Emphasize the benefits	Personal gain/altruism Young people generally
Avoid further “stigmatizing” young people with T1D	Ups and downs are normal part of living with a chronic illness
Tell us who you are	Provide background information on the research team
Sources of mental health support	
Internet‐based services	Anonymity/privacy Convenience “Low intensity” interventions may facilitate further help seeking
Peer support	Opportunity to connect with others Assist with “normalizing the struggle”
Face‐to‐face treatment	Need to understand diabetes Tailored to a young person's needs

Almost overwhelmingly, participants emphasized the need for better education for the community generally, people with type 1 diabetes and diabetes health professionals about the link between mental and physical health in type 1 diabetes. They felt that raising awareness would assist with:

- “normalizing” the emotional struggles that many young adults with type 1 diabetes face,
- facilitating more open and “comfortable” discussion about mental health issues (by young adults and health‐care providers),
- emphasizing the importance and personal relevance of mental health research; and
- making research participation a less stigmatizing and more acceptable option for young adults with type 1 diabetes.

And then you think maybe if we talked about this amongst our little community more people wouldn't be afraid to talk about it. (Female, age 20, diagnosed at 11 years)

Many participants stressed the need for researchers to adopt innovative approaches to research promotion. While many agreed that social media (including Facebook and Twitter) are potentially a useful resource, the consensus was that this should be combined with dissemination strategies that engage positive role models (eg youth ambassadors, people with lived experience) and organizations and health professionals that young adults with type 1 diabetes trust.

I'd definitely be a lot more receptive if it [i.e., study invitation] came from a friend or someone I know personally [ ] If my educator or endocrinologist approached me I'd be open to it too, but only because I have a good working relationship with them. (Female, age 18, diagnosed at 15 years)

It was suggested that recruitment messaging should be “transparent” by providing sufficient detail upfront about the time commitment, the kind of information that will be sought from participants, how the data will be used to benefit young adults with type 1 diabetes, and if/how participants could access the research data. Participants also highlighted the importance of flexibility within research projects to accommodate young adults’ busy lifestyles.

But give us our own time to do it because it is pot luck when we're all free. (Female, age 21, diagnosed at 10 years)

It's much easier to set something up over the phone or over email [ ] if you ask somebody to come in they'd probably [ ] this generation is lazy so they wouldn't want to take the extra step. (Female, age 21, diagnosed at 6 years)

Participants generally agreed that mental health research was potentially a “hard sell” to young adults, due largely to stigma‐related concerns. Low tolerance was expressed for messaging that made explicit reference to diagnostic labels (eg “depression” and “anxiety”). Instead, they preferred a subtler approach that normalized emotional issues as part of the experience of living with type 1 diabetes. The following suggestions were offered as examples; “Does diabetes stress you out?,” “Feel like a mood tune‐up from time to time?”.

[ ] it is okay to feel down, it is okay to struggle, everyone occasionally will go through this. (Male, age 24, diagnosed at 19 years)

Involvement of young adults in both the design and oversight of the research process was suggested as important for ensuring the “legitimacy” of research studies in the eyes of young adults and for ensuring that research procedures appropriately reflected their needs. It was suggested that researchers seek nominations/applications for youth representation on project advisory or steering committees.

#### 3.2.4 Sources of mental health support

Young adults nominated 3 preferred sources of psychosocial support, namely peer‐supports, online tools and face‐to‐face counselling (See Table 3). Overwhelming, peer support was discussed as a means of facilitating connection with others and reducing feelings of isolation and loneliness. Both online and face‐to‐face peer support options were discussed as providing opportunities for young adults to share their diabetes experience without feeling judged, to seek opinion on practical issues and to obtain advice when required. “Chat rooms,” in particular, were mentioned as options for ensuring that peer support was provided in a way that ensured participant anonymity and privacy.

I think that peer support programs can be really good [ ] you can make connections and have friends who you know are also going through the same problems and who you know understand what you are feeling. (Female, age 22, diagnosed at 20 years)

Online and unguided (ie no human support) were described as providing the benefits of anonymity, privacy and convenience. However, the extent to which they could be sufficiently supportive and able to assist with “serious” emotional issues was questioned by some participants, who fell that these tools were generally impersonal and incapable of responding empathically and with encouragement to a young person's unique emotional needs. There was greater confidence in digital tools that included human contact in the form of peer support, mentoring or therapist support, for example chat rooms, discussion boards and Skype facilitated therapy. It was further suggested that these tools might have the advantage “breaking the ice” for someone in need of more structured face‐to‐face therapy.

It's helpful to hear a voice behind it [ ] not just see a screen with information on it. That's how I am in terms of websites. (Female, age 20, diagnosed at 9 years)

There was a unanimous preference for support for serious emotional difficulties to be provided in the face‐to‐face context. Participants made it clear, however, that mental health professionals needed to be educated about diabetes per se, as well as the link between the emotional and physical health outcomes in type 1 diabetes.

[ ] if they are not completely educated about diabetes, you do a lot more explaining than getting answers. I go in there and it's like ‘I should have stayed at home, I could have done this myself. (Female, age 21, diagnosed at 7 years)

## 4 DISCUSSION

Findings from this qualitative study suggest that attitudes held by young adults with type 1 diabetes towards mental health are complex. Comments made by our participants suggest that this group conceptualizes the relationship between emotional struggles and type 1 diabetes in a nuanced way. While many participants reported feeling stressed, lonely, isolated, hopeless, ashamed, guilty and sad from time‐to‐time, these were discussed as side‐effects of living with a chronic and complex disease, in other words “normal,” and not as mental health issues per se.

The use of diagnostic labels to reference psychological distress within the context of diabetes, such as depression and anxiety, was met with some resistance by our participants. One possibility is that this represents an “externalizing” (ie beyond my control) of emotional distress, which reflects either a conscious or unconscious effort on the part of young adults to reduce externally imposed stigma and internally constructed self‐stigma. Indeed, attribution theory predicts that external causal predictions of negative situations generate a more positive self‐outlook than internal attributions.^27^, ^28^ At the same time, these findings provide support for the view that emotional distress in diabetes is more appropriately considered a continuous, scalable psychological characteristic (ie diabetes distress) rather than a discrete comorbid clinical condition (eg depression).^29^

Decision making regarding mental health research was found to be multifaceted and influenced by strong cognitive, behavioural and emotional elements. Cognitive considerations included the personal relevance of mental health studies, understanding of research processes and awareness of research opportunities. Behavioural factors encompassed the impact of research protocols on young adults’ daily activities and schedules. Affective elements included perceived stigma and self‐stigma, feelings of altruism and the desire to “make a difference” and trust in the research team. These findings concur with previous research highlighting the complexity of research participation decision making.^20^, ^21^, ^22^ Moreover, they imply that focussed attention to increase investment and confidence in and understanding of mental health research is essential for enrolling and retaining young adults with type 1 diabetes in mental health studies.

The responses of participants confirmed the viability of social media and Internet outreach for recruitment of young adults with type 1 diabetes.^19^ They also identified a number of advertisement features that might impact ad success including, graphics (eg the use of “youth ambassadors”), language (eg transparent and de‐stigmatizing) and placement (eg Facebook and trusted organizations/advocacy groups). Interestingly, face‐to‐face approaches involving direct referral by health‐care providers were also recommended, despite respondents’ concerns about their own comfort and the willingness of treating specialists to discuss emotional issues. Poor communication between young diabetes patients and health‐care providers about emotional issues and coping strategies has been documented previously.^6^ Interventions aimed at equipping health‐care providers with sufficient skills and information to confidently discuss psychosocial issues with their patients may not only facilitate study recruitment, but also improve clinic attendance.

Contrasting with the view that young adults prefer digital tools over more traditional methods of psychosocial support, participants in this qualitative study expressed a strong preference for human and/or therapist support for emotional issues. This finding is in line with the outcomes of studies showing that many young Australians show a strong preference for face‐to‐face help.^30^, ^31^ Of particular concern was the capacity of digital tools to provide personalized and empathic support. We have recently published data showing that there is potential for adults to experience positive therapeutic alliance features (eg including bond, trust, empathy, collaboration and openness) in the online, unguided support environment.^32^ Further research is required to investigate systematically young adults’ perceptions and experience of non‐specific features of therapeutic encounters in digital mental health interventions.

### 4.1 Limitations

Our recruitment methods yielded a group of mildly distressed predominantly female volunteers with type 1 diabetes. Therefore, our findings cannot be assumed to relate to more distressed young adults, those less willing to take part or to males. Young adult males present a recruitment challenge to mental health researchers,^9^, ^10^, ^11^, ^12^ and there are likely to be attitudinal and experiential factors specific to young men with type 1 diabetes that were not captured in our data. Future qualitative studies may benefit from sampling young men using methods with demonstrated effectiveness in research with other groups that are difficult to involve (eg low incidence and stigmatized groups). For example, snowball sampling is especially useful for research on sensitive topics, where some degree of trust is needed to initiate the recruitment process.^33^

Another limitation relates to the unfamiliarity of most participants with mental health research. As young adults’ reflections are likely to be grounded in day‐to‐day experience, the applicability of our findings to a more “experienced” group of participants remains to be determined.

### 4.2 Implications

Based on the findings of this qualitative study, we can offer the following broad suggestions for health professionals and mental health researchers regarding young patients with type 1 diabetes:

Health professionals:

- Continue to develop and implement strategies aimed at improving recognition, discussion and management of the emotional needs of young adults with type 1 diabetes in routine care.
- Avoid “overpathologizing” the emotional struggles of young adults with type 1 diabetes.

Mental health researchers:

- Adopt a participatory research approach that prioritizes involvement of young adults in all stages of the research process, including protocol development, recruitment messaging and activities, and dissemination of research findings. This may include providing opportunities for involvement as co‐researchers and co‐authorship in scientific manuscripts.
- Online and social media strategies that take advantage of a study's inclusion criteria (eg age, gender and location) and provide a “click‐through” option for screening and enrolment are a palatable option to young adults.
- As part of a two‐pronged recruitment strategy, preface and/or accompany recruitment activities targeting young adults with adjunctive campaigns aimed at educating stakeholder groups, health professionals and other potential sources of direct referral about the study.
- Carefully consider the content of recruitment messages. Wording of recruitment materials should reflect young adults’ conceptualization of distress as normative in diabetes. Pilot message content and formats with young adults prior to dissemination. Messaging that includes diagnostic labels (eg depression and anxiety) and/or potentially stigmatizing wording is likely to be perceived as personally irrelevant and potentially pejorative. Inclusion of testimonials from and images of “youth ambassadors” is likely to be helpful.
- Online and print materials should clearly identify and personalize the research team by providing all relevant biographic details (including a photograph, qualifications and expertise) and contact information. If possible, provide an opportunity to meet the research team in person (eg seminar) or via video presentation (eg webinar).
- In addition to meeting the minimum requirements for standard informed consent, study materials should outline clearly the processes by which the unique needs of young adults have been considered in the research protocol, including details of youth involvement and pilot testing.
- There was limited evidence that offering incentives for participation would boost recruitment; however, young adults reported that they would be motivated by opportunities to learn about themselves and to help others. Clearly specifying the potential personal and broader impacts of the research process and study findings.
- Young adults expressed genuine interest in engaging with others with type 1 diabetes. As far as possible, provide opportunities for young adults with diabetes to connect with their peers, either directly (eg focus groups) or indirectly (eg online), throughout the research process.

Future research testing the impact of these recommendations on study recruitment is warranted.

## 5 CONCLUSION

Effective provision of mental health support for young adults with type 1 diabetes requires rigorous testing of psychological treatments. Unfortunately, recruitment of this cohort is difficult, and small sample sizes are often an issue. Understanding the critical factors that promote engagement in this type of clinical research is an important step in overcoming recruitment barriers. Our qualitative exploration of the attitudes of young adults with type 1 diabetes to their mental health and mental health research has provided valuable insights into the complex dynamics of engaging and retaining this vulnerable population in mental health studies.

## CONFLICT OF INTEREST

None declared.

## References

[1] Australian Bureau of Statistics . National Survey of Mental Health and Wellbeing: Summary of Results. Canberra, ACT: Australian Bureau of Statistics; 2007.

[2] de Wit M , Snoek FJ . Depressive symptoms and unmet psychological needs of Dutch youth with type 1 diabetes: results of a web‐survey. Pediatr Diabetes. 2011;12(3 Pt 1):172‐176. 20561242 10.1111/j.1399-5448.2010.00673.x

[3] Harris MF , Jayasinghe UW , Chan BC , et al. Patient and practice characteristics predict the frequency of general practice multidisciplinary referrals of patients with chronic diseases: a multilevel study. Health Policy. 2011;101:140‐145. 21126795 10.1016/j.healthpol.2010.10.019

[4] Bernstein CM , Stockwell MS , Gallagher MP , Rosenthal SL , Soren K . Mental health issues in adolescents and young adults with type 1 diabetes: prevalence and impact on glycemic control. Clin Pediatr (Phila). 2013;52:10‐15. 22988007 10.1177/0009922812459950

[5] Bryden KS , Dunger DB , Mayou RA , Peveler RC , Neil HA . Poor prognosis of young adults with type 1 diabetes: a longitudinal study. Diabetes Care. 2003;26:1052‐1057. 12663572 10.2337/diacare.26.4.1052

[6] Australia D . Young Adults with Diabetes Needs Analysis. Melbourne: National Diabetes Services Scheme; 2006.

[7] Cunradi CB , Moore M , Killoran G , Ames G . Survey nonresponse bias among young adults: the role of alcohol, tobacco and drugs. Subst Use Misuse. 2005;40:171‐185. 15770883 10.1081/ja-200048447

[8] Galea S , Tracy M . Participation rates in epidemiological studies. Ann Epidemiol. 2007;17:643‐653. 17553702 10.1016/j.annepidem.2007.03.013

[9] Ellis L , Collin P , Davenport T , Hurley P , Burns JM , Hickie IB . Young men, mental health, and technology: implications for service design and delivery in the digital age. J Med Internet Res. 2012;14:e160. 23171827 10.2196/jmir.2291 PMC3510732

[10] Ellis L , Collin P , Hurley P , Davenport T , Burns JM , Hickie IB . Young men's attitudes and behaviour in relation to mental health and technology: implications for the development of online mental health services. BMC Psychiatry. 2013;13:119. 23601273 10.1186/1471-244X-13-119 PMC3651363

[11] Ellis L , McCabe K , Rahilly K , et al. Encouraging young men's participation in mental health research and treatment: perspectives in our technological age. Clin Investig (Lond.). 2014;4:881‐888.

[12] Forgarty A , Proudfoot J , Whittle E , et al. Preliminary evaluation of a brief web and mobile phone intervention for men with depression: men's positive coping strategies and associated depression, resilience and work and social functioning. JMIR Ment Health. 2017;4:e33. 28798009 10.2196/mental.7769 PMC5571234

[13] Krischer J , Cronholm P , Burroughs C , McAlear CA , Borchin R , Easley E . Experience with direct‐to‐patient recruitment for enrollment into a clinical trial in a rare disease: a web‐based study. J Med Internet Res. 2017;19:e50. 28246067 10.2196/jmir.6798 PMC5350442

[14] White M , O'Connell M , Cameron F . Clinical attendance and disengagement of young adults with type 1 diabetes after transition of care from paediatric to adult services (TrACed): a randomised, open‐label, controlled trial. Lancet Child Adolesc Health. 2017;1:274‐283. 10.1016/S2352-4642(17)30089-5 30169183

[15] Gorman J , Roberts S , Dominik S , Malcarne V , Dietz A , Su I . A diversified recruitment approach incorporating social media leads to research participation among young adult‐aged female cancer survivors. J Adolesc Young Adult Oncol. 2014;3:59‐65. 24940529 10.1089/jayao.2013.0031 PMC4048966

[16] Fenner Y , Garland S , Moore E , et al. Web‐based recruiting for health research using a social networking site: an exploratory study. J Med Internet Res. 2012;14:e20. 22297093 10.2196/jmir.1978 PMC3374531

[17] Ramo D , Rodriguez T , Chavez K , Sommer M , Prochaska J . Facebook recruitment of young adult smokers for a cessation trial: methods, metrics and lessons learned. Internet Interv. 2014;1:58‐64. 25045624 10.1016/j.invent.2014.05.001 PMC4100621

[18] Burns J , Birrell E . Enhancing early engagement with mental health services by young people. Psychol Res Behav Manag. 2014;7:303‐312. 25473320 10.2147/PRBM.S49151 PMC4251759

[19] Balfe M , Doyle F , Conroy R . Using facebook to recruit young adults for qualitative research projects: how difficult is it? Comput Inform Nurs. 2012;30:511‐515. 23079479 10.1097/NXN.0b013e31826e4fca

[20] Newington L , Metcalfe A . Factors influencing recruitment to research: qualitative study of the experiences and perceptions of research teams. BMC Med Res Methodol. 2014;14:10. 24456229 10.1186/1471-2288-14-10 PMC3903025

[21] Miller SM , Hudson SV , Egleston BL , et al. The relationships among knowledge, self‐efficacy, preparedness, decisional conflict, and decisions to participate in a cancer clinical trial. Psychooncology. 2013;22:481‐489. 22331643 10.1002/pon.3043 PMC3374030

[22] Treweek S , Lockhart P , Pitkethly M , et al. Methods to improve recruitment to randomised controlled trials: Cochrane systematic review and meta‐analysis. BMJ. 2013;3:e002360. 10.1136/bmjopen-2012-002360 PMC3586125 23396504

[23] Frew PM , Hou S‐I , Davis M , et al. The likelihood of participation in clinical trials can be measured: the Clinical Research Involvement Scales. J Clin Epidemiol. 2010;63:1110‐1117. 20303711 10.1016/j.jclinepi.2009.12.002 PMC2892193

[24] Kroenke K , Spitzer RL . The PHQ‐9: a new depression diagnostic and severity measure. Psychiatr Ann. 2002;32:509‐515.

[25] Fisher L , Glasgow RE , Mullan JT , Skaff MM , Polonsky WH . Development of a brief diabetes distress screening instrument. Ann Fam Med. 2008;6:246‐252. 18474888 10.1370/afm.842 PMC2384991

[26] Clarke V , Braun V . Thematic analysis In: Teo T, ed. Encyclopedia of Critical Psychology. New York, NY: Springer New York; 2014:1947‐1952.

[27] Corrigan PW . Mental health stigma as social attribution: implications for research methods and attitude change. Clin Psychol Sci Pract. 2000;7:48‐67.

[28] Weiner B . Judgments of Responsibility: A Foundation for a Theory of Social Conduct. New York, NY: Guilford Press; 1995.

[29] Fisher L , Gonzalez J , Polonsky W . The confusing tale of depression and distress in patients with diabetes: a call for greater clarity and precision. Diabet Med. 2014;31:764‐772. 24606397 10.1111/dme.12428 PMC4065190

[30] Burns JM , Davenport TA , Durkin LA , Luscombe GM , Hickie IB . The internet as a setting for mental health service utilisation by young people. Med J Aust. 2010;192:S22. 20528703 10.5694/j.1326-5377.2010.tb03688.x

[31] Bradford S , Rickwood D . Adolescent's preferred modes of delivery for mental health services. Child Adolesc Mental Health. 2014;19:39‐45. 10.1111/camh.12002 32878356

[32] Clarke J , Proudfoot J , Whitton A , et al. Therapeutic alliance with a fully automated mobile phone and web‐based intervention: secondary analysis of a randomized controlled trial. JMIR Ment Health. 2016;3:e10. 26917096 10.2196/mental.4656 PMC4786687

[33] Shaghaghi A , Bhopal R , Sheikh A . Approaches to recruiting ‘heard‐to‐reach’ populations into research: a review of the literature. Health Promot Perspect. 2011;1:86‐94. 24688904 10.5681/hpp.2011.009 PMC3963617

